# Secondary use of genomic data: patients’ decisions at point of testing and perspectives to inform international data sharing

**DOI:** 10.1038/s41431-023-01531-5

**Published:** 2024-03-25

**Authors:** Melissa Martyn, Emily Forbes, Ling Lee, Anaita Kanga-Parabia, Rona Weerasuriya, Elly Lynch, Penny Gleeson, Clara Gaff

**Affiliations:** 1 Murdoch Children’s Research Institute, The Royal Children’s Hospital, 50 Flemington Road, Parkville, VIC 3052 Australia; 2 Melbourne Genomics Health Alliance, Parkville, VIC 3052 Australia; 3 Department of Paediatrics, University of Melbourne, Parkville, VIC 3052 Australia; 4 Victorian Clinical Genetics Services, Parkville, VIC 3052 Australia; 5 Deakin Law school, Deakin University, Burwood, VIC 3125 Australia

**Keywords:** Genetic counselling, Health policy, Ethics, Genetic testing, Genetics research

## Abstract

International sharing of genomic data files arising from clinical testing of patients is essential to further improve genomic medicine. Whilst the general public are reluctant to donate DNA for research, the choices patients actually make about sharing their clinical genomic data for future re-use (research or clinical) are unknown. We ascertained the data-sharing choices of 1515 patients having genomic testing for inherited conditions or cancer treatment from clinical consent forms. To understand the experiences and preferences of these patients, surveys were administered after test consent (RR 73%). Almost all patients (98%) consented to share their data. Survey respondents’ decision recall was high (90%), but poorer if English was an additional language (*p* < 0.001). Parents deciding on behalf of children were over-represented amongst data-sharing decliners (*p* = 0.047) and decliners were more likely to believe that stored data could be easily reidentified (*p* < 0.001). A quarter of respondents did not know if reidentification would be easy and 44% of them were concerned about this possibility. Of those willing to share data overseas (60%), 23% indicated the recipient researcher’s country would affect their decision. Most respondents (89%) desired some ongoing control over research use of their data. Four preliminary data-sharing profiles emerged; their further development could inform tailored patient resources. Our results highlight considerations for establishment of systems to make clinical genomic data files available for reanalysis locally and across borders. Patients’ willingness to share their data – and value of the resulting research – should encourage clinical laboratories to consider sharing data systematically for secondary uses.

## Introduction

The need for better diagnostic yield and tailored patient management is driving the increasing availability of genomic sequencing in healthcare systems around the world [1]. International sharing of genomic data - primarily that generated in research - has contributed to many advances in clinical genomics, including the development of analytic tools [discussed in [2]]. Genomic data sharing remains essential for further improvement in genomic medicine. With estimates that over 60 million patients will have had genomic sequencing as part of their healthcare by 2025 [3], the value of data generated by medical laboratories performing testing for the primary purpose of clinical care (‘clinical genomic data’) is considerable. In the literature on genomic data sharing, little to no distinction is made between data arising from clinical care and that generated by research. Furthermore, in some health systems - like Australia’s – clinical genomic data is under the governance of distinct entities, e.g. the pathology laboratory which generated the data. This means sharing of clinical genomic data files may be required for clinical re-analysis (e.g. for a new clinical presentation). Yet, sharing data for secondary clinical re-analysis is rarely distinguished from sharing for research. These distinctions matter, as the regulation of research and healthcare are fundamentally different [3], with healthcare legislation and standards focused on the provision of safe, high-quality care and protection of privacy [4]. The regulatory environment in which clinical genomic data is situated is therefore distinct from that governing sharing of research genomic data.

Most studies on genomic data sharing to date have investigated participants in genomic/biobanking research and/or the general public [5–8]. There are several reasons why these results cannot be extrapolated to patients having clinical genomic testing for diagnosis, prognosis and management of a medical condition. In contrast to clinical testing, where consent for researcher access to data is optional (e.g. UK NHS Genomic Medicine Service), research projects involving genomic data collection commonly require participants’ agreement to share that data [e.g. 100,000 Genomes project; [9] ClinSeq [10]] and so do not capture the attitudes of those unwilling to share. Community attitudes towards genomic data sharing are an important consideration when establishing data governance of clinical genomic systems [see for e.g. [11]], but general public and patient views on data sharing may differ, for example, due to differences in knowledge of genomics [6] or increased awareness of the benefits of data sharing [12] and so are not necessarily representative of those whose clinical genomic data will be stored and shared. The views of patients undergoing genomic testing in healthcare are critical to inform governance, policies and procedures for the sharing of clinical genomic sequencing data – where data is held in accordance with clinical regulatory requirements - yet evidence of patient views is limited [12]. The sharing of clinical genomic data files is not a hypothetical scenario. One example is a clinical platform [13] used by medical testing laboratories in Australia to support them to independently store, analyse, interpret and report patient genomic tests; the primary purpose of this data system is clinical, but it also supports the sharing of genomic data files for secondary clinical and research use.

To address the gap in knowledge needed to inform policy-making, we investigate the actual data-sharing decisions of patients when consenting to genomic sequencing for clinical care. Post-counselling, we surveyed patients to determine their understanding and their recall of their data-sharing decision. We also explored survey respondents’ perceptions of and attitudes towards clinical genomic data management and sharing.

## Materials and methods

### Study design and setting

This was a mixed methods study conducted as part of The Melbourne Genomics Health Alliance program. This study has Human Research Ethics Committee approval from Melbourne Health (13/MH/326). All participants provided informed consent for clinical testing and for participation in research activities.

### Participants, pre-test counselling, and consent

From 2016-2020, Melbourne Genomics Health Alliance evaluated the utility of diagnostic and treatment-directed genomic testing for a range of rare disease and cancer indications [14]. Patients were offered research-funded genomic testing within clinical care: exome sequencing was performed for all conditions on germline and, where relevant, tumour DNA, except for solid tumours, where a ~ 400 gene multi-gene pan-cancer panel was used.

Adult patients (*n* = 988) and parents of paediatric patients (*n* = 527) gave consent for genomic testing after pre-test genetic counselling. This counselling was provided by professional genetic counsellors for all indications except solid cancers, where a medical oncologist with genomics expertise performed pre-test counselling. Genetic counselling included discussion of data sharing; health professionals undertaking consent discussions were provided with a guide to ensure consistent information provision (Supplementary File 1). Of the 219 individuals/families who spoke English as an additional language, 41% used an interpreter during the consent process (6% of the total cohort). By giving consent for testing, patients agreed their genomic data may be shared in a way that does not identify them (‘anonymised’). Patients had the choice to opt in to sharing genomic data that is re-identifiable for a range of future activities, with examples provided (Supplementary File 2). Clinical consent forms documented this data-sharing decision.

### Data collection –clinical care

Patients’ data-sharing decisions were documented on the clinical consent form and entered into a REDCap electronic data capture tools database hosted at the Murdoch Children’s Research Institute [15]. All consent forms were audited to check the accuracy of REDCap records after the completion of recruitment.

Sociodemographic information: gender; age; postcode (enabling socioeconomic and location inferences); whether first language was English and country of birth; Aboriginal and/or Torres Strait Islander identity were collected for all patients through clinical care and documented in the study database.

### Data collection – research survey

Patients and parents of paediatric patients who consented to testing were invited to complete a survey after pre-test counselling but prior to return of their genomic test results. Surveys were available in printed or online formats in English. Patients who used an interpreter or who indicated other barriers to completing surveys were offered the choice of completing the survey by telephone with a research assistant and, where necessary, an interpreter. The survey was not administered if the person tested had died, if results were returned before the survey could be sent (e.g. after rapid testing of acutely unwell patients), to those who declined to participate in surveys, or if interpretation was needed and it could not be arranged. Furthermore, surveys were not administered to families offered genomic testing as part of perinatal autopsy, but their data-sharing choices are reported.

Surveys used categorical and open-ended questions to collect data on: the actual data-sharing decision, including open ended questions on information provision and concerns; knowledge of the uses of stored data; and hypothetical preferences for data storage and sharing in the future. Questions and response options for the latter were developed by the study team, based on questions and responses from a focus group study (Gleeson, unpublished) which were converted to closed questions with categorical response options and space provided for open comments. Questions explored models of consent, concerns and beliefs about the ease of re-identification, and preferences for sharing with different types of organisations. The main reasons for a respondent’s data-sharing decision were also collected using an open-ended question but are not reported here.

### Statistical analysis

Quantitative data were analysed using descriptive statistics in Stata/IC version 15.0 [16]. Fishers exact or Chi-squared tests were used to assess differences for independent categorical variables, depending on group size. Multivariable logistic regressions were conducted to identify potential demographic characteristics associated with survey response and recall of data-sharing decision. Due to collinearity between variables (e.g. English as an additional language and country of birth), or a small number of observations (e.g. identifying as Aboriginal and/or Torres Strait Islander), some parameters were excluded from the model. Mann-Whitney U tests were used to assess continuous data. P-values are reported for results where *p* < 0.05.

Informed by Shabani et al. [7], directed inductive content analysis [17] was used to analyse open-ended responses. Comments made by respondents to open-ended questions did not always relate to the questions posed; author RW thus initially coded all open-ended responses to key concepts of interest covered by the open questions. For example, all comments relating to remaining concerns about data sharing were grouped, no matter which open comment box they were written in. Authors RW and MM then carried out iterative rounds of inductive coding. The initial round was influenced by, but not restricted to, the factors identified in a systematic review [7]. Differences in interpretation were resolved through discussion. Insights drawn from the data and presented here were refined through repeated examination and discussion with a wider group of evaluation researchers across the Melbourne Genomics Health Alliance.

Qualitative comments and attitudes to data sharing were further analysed across an individual’s response to all survey questions to draw out themes or patterns consistent across patients’ responses, using methods informed by Pruitt and Adlin’s [18] description of persona development for product design. This information was analysed to detect psychographic factors influencing responses to identify skeletal ‘profiles’ [18] (i.e. people who showed common attitudes, interests, responses, or values similar to or distinct from other sub-groups of patients).

## Results

### Patient data sharing consent choices

When giving consent for clinical genomic sequencing, almost all patients (98%; 1480/1515) agreed to share their (re-identifiable) data for research (Supplementary File 2), with 35 declining to share (‘decliners’). Parents of paediatric patients were overrepresented in the decliners group (18/35 v 509/971, *p* = 0.047, Fisher’s exact). The decliner group did not differ from the overall cohort by: malignant or hereditary condition; Australian or overseas born; socio-economic status. As noted in methods, consent for anonymous sharing was a condition of testing.

### Survey respondent demographics

In total, 1408 surveys were administered (including to 31 decliners). A total of 1030 responses were received (73% response rate), including 16 from the decliners group (52% response rate). Surveys were returned a median of 32 days (IQR 8–71) after respondents agreed to clinical testing and signed the consent form. The demographics of survey respondents are shown in Supplementary File 3. Six per cent of survey respondents used assistance to complete surveys (i.e. telephone interpreter). Compared to the overall survey frame (those sent surveys), survey respondents were significantly more likely to be: adult patients (adjusted odds ratio [OR] 1.7); speak English as a first language (OR 1.8); from the higher quintiles of socioeconomic status (Fourth-quintile OR 1.63 Fifth-quintile OR 1.57, (Supplementary File 3).

### Pre-test counselling

Discussing data sharing for research is one aspect of pre-test counselling for clinical genomic testing, which aims to ensure people understand the possible outcomes of testing and have considered the potential implications for themselves and other family members. The majority of respondents (96%; 954/997) reported receiving enough information about data sharing during pre-test counselling. Decliners and those whose first language was not English were more likely to report they had not received enough information (19% v 4% *p* = 0.028; 11% v 3% *p* = 0.001; Fisher’s exact).

During pre-test counselling, patients were informed that their data can be shared in an ‘anonymised’ format for the purpose of advancing knowledge generally; the survey question on this was correctly answered by the majority of respondents (89%, 912/990), with 1% (*n* = 12) answering incorrectly and the remainder unsure. Moreover, most respondents accurately recalled the decision they made regarding sharing of their re-identifiable data. Only 1% (11/1004) recalled incorrectly: five decliners stated they had consented; six who had consented, believed they had declined. In addition, 6% (*n* = 66) could not recall their decision. The median response time for those who recalled their decision incorrectly or were unsure of their decision was twice as long as the response time for those who correctly recalled their decision (62 days vs. 29 days, *p* < 0.001; Mann-Whitney U test). Those who did not correctly recall their data-sharing decision were more likely to speak English as an additional language [OR = 3.11; 95% confidence interval (CI) 1.72–5.65; *p* < 0.001 after adjusting for gender and participant type (adult or child) by multivariable logistic regression].

### Concerns about data sharing

A minority of respondents (8%; 78/988) had remaining concerns about data sharing or were uncertain after pre-test counselling; although most of these people (94%, 73/78) did go on to consent to data sharing. Those with remaining concerns were over-represented among those who indicated they did not receive enough information about data sharing (36% vs. 7%; 15/42 vs. 62/942; *p* < 0.001).

Forty-five respondents made comments relating to concerns about data sharing (Table 1). In addition to concerns about security and the potential implications of data sharing for access to insurance, some respondents would have valued additional information on data-sharing processes. Only 6 of the 16 decliner respondents made comments about their decision, so distinctions between them and other respondents cannot be made (Supplementary File 4).

**Table 1:** Remaining concerns about data sharing (*n* = 52 comments from 45 respondents).

Theme	Example Quote
**Use and Potential ramifications (*n*** = **19**^**a**^)	
Insurance (*n* = 13)	I am not sure how the data sharing would affect my insurance including disability fund and policies. (Hereditary, Adult)
Employment (*n* = 4)	If legislation will change and other organisations will be able access genetic test results impacting on my son’s personal options, e.g., employment /insurance (Hereditary, Parent)
**Security, confidentiality and privacy (*n*** = **12)**	
	I have concerns about the ability to keep the information private and confidential (Hereditary, Parent).
	Do you have adequate cyber security to avoid hackers obtaining my sensitive information. Privacy breach. (Hereditary, Adult)
	Ability to link data to donor (Hem/Malignant, Adult)
**Data sharing processes (*n*** = **14)**^**b**^	
Seeking additional information (*n* = 9)	How do you share information? Like through the website, internet, or how do you share? (Hereditary, Adult)
	the sanitisation and de-identification of data and sharing processes are unclear, including scope, agencies allowed, etc. (Hereditary, Parent).
Notification and ability to approve requests (*n* = 4)	I think it’s a good idea whenever someone’s data is shared, he/she should be notified automatically (Hem/Malignant, Parent)
**Could not recall information provided (*n*** = **2)**	I can’t remember who the data will actually be shared with. Had too much information to take in at once. (Hereditary, Adult)
**Research inconsistent with personal preferences (*n*** = **2)**	I only want to help cancer research. I don’t want me or my family genes shared for any other purpose (Hem/Malignant, Adult)
	From a personal point of view, I do not agree with abortion or termination of IVF fetus or children naturally conceived. If this research showed and gave people the option to terminate a child because they had a medical condition, I would not be happy to be a part of that study or enabling that option. (Hereditary, Adult)
**Discomfort with the unknown (*n*** = **3)**	… some uneasiness with the unknown. (Hereditary, Parent)

^a^2 responses not represented in examples shown.

^b^1 response not represented in examples shown.

No differences were noted in response to the pre-test counselling questions between patients with rare disease, who received pre-test counselling from qualified genetic counsellors, and those with cancer, who were counselled by a trained oncologist.

### Perceptions of re-identification

We asked patients how difficult they thought it would be for someone to be identified from their stored genome sequence, and how concerned they would be if they were identified. Eighteen per cent of those who consented to data sharing and 54% of decliners believed it would be easy to be re-identified from their stored data (*p* < 0.001). Sixty per cent of respondents said they would be unconcerned if they were re-identified from stored data. Respondents with suspected hereditary conditions [χ² (2, *n* = 909) = 7.28; *p* = 0.007], encompassing those having a sequencing test [χ² (2, *n* = 909) = 5.82; *p* = 0.015] were significantly more likely to be concerned about being identified from their data than those with haematological/malignant conditions, which includes those having a cancer panel test.

The 683 people who gave a definitive answer to both questions were grouped based on their perceptions of ease of identification and their concern if identified (Table 2). Importantly, an additional 223 people did not know how easy it would be to identify a person from their stored data; these were split between concerned (*n* = 99) and unconcerned (*n* = 124). Twenty-one of those 223 people provided relevant comments; some reflected the lack of information provided about security and privacy measures, while others indicated trust in current measures but some concern about future risk “as technology advances”.

**Table 2:** Perception of ease of identification from stored data and level of concern if re-identified from stored data (excluding respondents who answered ‘unsure’).

	Concerned	Unconcerned	Total
**Difficult**	208 (30.5%)	310 (45%)	518
**Easy**	56 (8%)	109 (16%)	165
**Total**	264	419	683

### Preferences for future data sharing consent models

Survey respondents chose between five different models of consent for future data sharing. The majority (89%; 791/893) selected an option indicating a desire for some degree of ongoing control over use of their data in research; the most frequent answer was that the participant should initiate contact if they wished to opt out of research (38.5%; 343/893). Half (50%; 448/893) wished to be notified regarding each new research study, with responses split evenly between preferring to opt in (25%; 222/893) or opt out (25%; 226/893) each time. Of the 13 (1.5%) respondents who indicated they would not give consent for their data to be stored and reused for research under any circumstances, 12 had provided consent for future sharing of their data on their clinical consent form.

### Attitudes towards sharing with potential data recipients

Respondents indicated whether and to what extent they agreed with researchers at certain types of organisations undertaking two activities: accessing and using their stored genomic data for research purposes; and re-identifying people from their stored data. Respondents were significantly more likely to agree with access and use of genomic data by researchers at Australian not-for-profit organisations than not-for-profit organisations abroad (*p* < 0.001). Two-thirds (61%; 319/527) of those who had indicated they would agree to allow researchers at not-for-profit organisations outside Australia to access their data said the recipient researcher’s country did not matter, but one quarter (23%; 119/527) said it did, with 89 respondents unsure. Further detail is shown in Table 3. Of the 16 decliners, 11 would share data with members of the Alliance, 5 with researchers at Australian not-for profit organisations, and 1-2 would share with the other types of organisations listed.

**Table 3:** Respondents who agreed or strongly agreed with organisations accessing and using their genomic data and being able to re-identify individuals from their stored data.

Type of Organisation	Agree with access and use of genomic data	Agree with ability to re-identify
Members of the Alliance#	868/908 (96%)	695/899 (77%)
Researchers at Australian not-for-profit organisations	699/899 (78%)	379/895 (42%)
Researchers at not-for-profit organisations abroad	537/892 (60%)	269/895 (30%)
Researchers at pharmaceutical companies	484/894 (54%)	248/893 (30%)
Government	375/888 (42%)	238/881 (27%)
Researchers in other industries	315/887 (36%)	177/893 (20%)

#researchers at organisations that are members of the Melbourne Genomics or Australian Genomic Health Alliances.

Respondents’ willingness for their data to be shared outside Australia generally related to the use of the data (i.e., purpose of sharing), perceptions of systems and processes in place e.g. the country’s research governance, legal, and regulatory system, and similarity to/allegiances with Australia) (Table 4).

**Table 4:** Willingness for overseas (non-Australian) sharing or use of genomic data (*n* = 155 comments from 127 respondents).

Theme	Quote
**Purpose (*n*** = **31)**	
	Regarding overseas [non-Australian] research, pharma (local and overseas), government (local and overseas) and other companies (local and overseas) I would not immediately object to the use of the data as I would hope it’s for altruistic research regardless of location. I would like to know for what purpose genomic data is being used by foreign governments and companies (especially non-medical or pharma) is. (Hereditary, Adult) Doesn’t depend on where they are; it depends on what they want to use it for. (Hereditary, Parent,)
**Systems and Processes (*n*** = **63)**	
Ethical standards (*n* = 14)	Would need to ethically match Australian standards (Hereditary, Adult)
Privacy standards and data security (*n* = 22)	There is a great variability in the laws and regulations regarding; firstly data security, secondly the rights of data holders to share/sell data and thirdly the ability to patent genetic material. (Haem/Malignant, Adult,)
Legal frameworks (*n* = 7)	Depends what laws/regulations/processes/standards they have in place. (Hereditary, Adult) Countries that do not have anti-corruption processes in place would concern me. I would want to know the data was secure and we could not be tracked down. (Hereditary, Parent).
Approval processes (*n* = 4)	Assume there is some ‘rigour’ by which access is approved- Australia too (i.e., genuine research purposes) (Haem/Malignant, Adult) Only quality credible researchers should have access (Haem/Malignant, Adult)
Alliance/political structures (*n* = 14)	Countries with which Australia has an alliance; i.e., US, UK. (Hem/Malignant, Adult) Prefer democratically run countries over dictatorships etc (Haem/Malignant, Adult)
Health and medical research systems (n = 2)	Countries with Humanitarian and effective medical facilities and supported research programs (Hem/Malignant, Adult) Ethical Health systems e.g., Europe and some 3rd World. Not US (Hereditary, Adult).
**Uncertain (*n*** = **8)**	I am unsure whether I would be concerned depending on the country my data was being used by was (Hereditary, Parent)
**Mistrusting (*n*** = **17)**	Not interested in unfriendly countries (Haem/Malignant, Adult)
**Restrict to Australia (n** = **13)**	Do not wish for data sharing overseas (Hereditary, Adult)
**Unrestricted (*n*** = **23)**	I want as many people working together to try to make peoples lives better (Hereditary, Adult)

### Identification of preliminary ‘profiles’ of attitudes to data sharing

**Fig. 1:**
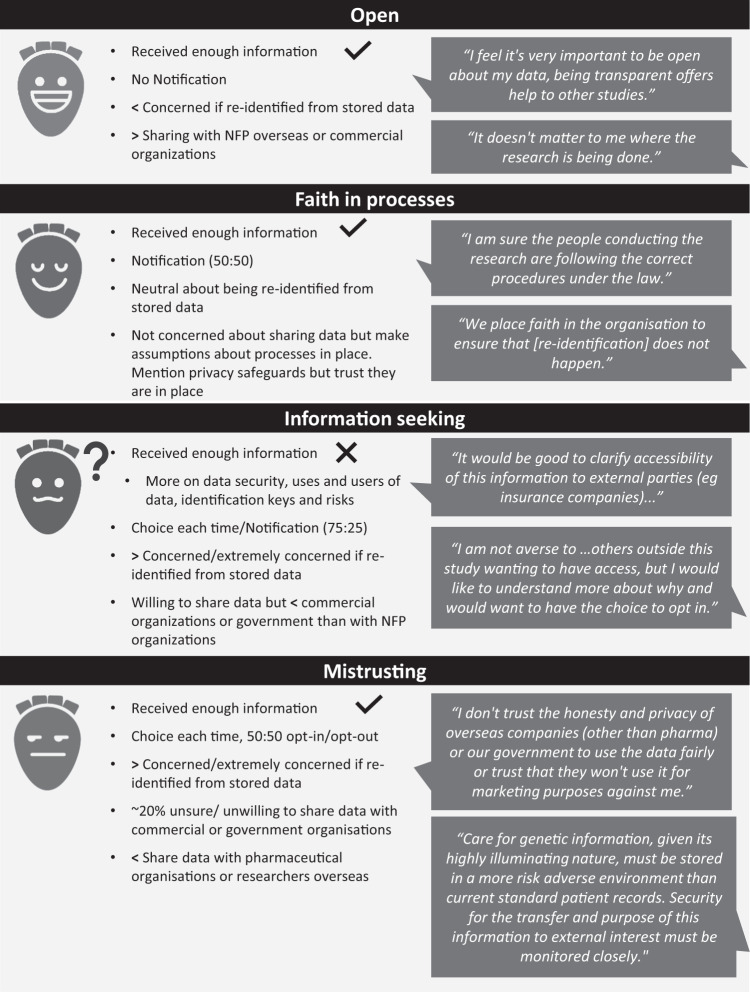
Profiles that represent decision-making styles, behaviours, needs, and goals concerning genomic data sharing. ‘Notification’/’Choice each time’ refs to preferences for future data sharing consent, as discussed in ‘preferences for future data sharing consent models’. NFP = ‘not-for profit’ organisations; ‘<’ = less likely; ‘>’ = more likely; ‘√ = likely to agree with statement; ‘X’ = likely to disagree with statement.

Using qualitative comments and quantitative survey data, we identified four preliminary (‘skeletal’) profiles (Fig. 1) representing decisions and attitudes to data sharing. t).

## Discussion

The success or failure of international efforts to share clinical genomic data across national boundaries [3] will ultimately rest on the decisions patients make. The prevalent attitude globally among the general public is unwillingness and uncertainty about donating anonymous DNA and health data for research [6]. In contrast, we found almost all patients opted-in to share their data (in a manner that enables re-identification) when consenting to their clinical genomic test. Data from 273 of these 1515 patients has in fact subsequently been made available for ethically approved research studies (D. Bodemer, personal communication). This suggests that patient uptake may not be a significant barrier to sharing clinical data for secondary uses. At the same time, our results also highlight areas to focus on to reduce the risk of patients declining clinical genomic testing (and thereby compromising their care), due to concerns about data sharing.

Although informed consent is a complex communication process involving more than just information provision [19, 20], information tailored to a patient’s situation is acknowledged as an important component of the consent process [21]. Information about data sharing has been shown to influence attitudes towards consent for data sharing of clinical genomic data [22]. In our study, a high number of respondents indicated they received enough information prior to making their decision. We do not claim that this reflects a high level of informed consent; there is no measure clinicians or researchers can use to be sure of genuine informed consent. The high level of satisfaction reported may be attributable to the opportunity patients had to ask questions specific to their situation during pre-test counselling. It cannot be assumed that this will continue to be the case as genomic testing is increasingly ‘mainstreamed’, that is, offered by medical specialists within the constraints of their existing consultations and with limited involvement from genetic specialists [23]. Although in our study we did not note any difference in satisfaction with information or remaining concerns between rare disease patients who received counselling from a qualified genetic counsellor and those with solid cancers counselled by a trained oncologist. Notably, decliners and people who did not speak English as a first language were over-represented in the group that reported not receiving enough information. Norstad and colleagues [24] also found participants from underserved and underrepresented populations were not well supported by informed consent processes to make informed choices. The challenge is to meet the immediate and ongoing needs of patients with diverse backgrounds, experiences, attitudes and values, while not overwhelming them.

In user-centred design of health applications, profiles have been used to bring the needs of different people into technological and software design [25]. An example in genomic healthcare is Genomics Advisor, a tool to support decision-making for genomic secondary findings, based on five ‘patient profiles’ identified from qualitative data [26, 27]. Information about data sharing could be similarly tailored, allowing patients an opportunity to acquire tailored information ahead of counselling, so that counselling can focus on issues relevant to their context and shared decision-making [26]. Our study is uniquely placed to inform such a tool as it explored patients’ real-life experiences of decision-making about sharing their clinical genomic data, as well their attitudes towards its re-use. Although not specifically designed to elucidate profiles, our results suggest four ‘skeletal profiles’ [18], which could be used to foreground information most relevant for informed decision-making. For example, ‘important points to consider’ could be prioritised for those very willing to share their data (i.e. ‘open’ skeletal profile). Further development of these skeletal profiles is needed, for example, elucidation of the ‘faith in processes’ and ‘mistrusting’ profiles found in our study could draw on work exploring factors underpinning different trust classes [28]. The inclusion of underserved communities in further profile development work will be needed to ensure they reflect clinical populations. A study specifically designed to validate the profiles and explore their utility is required.

There has been a move from broad opt-in/opt-out consent for research use to more granular options. Our concern that biobank and general public attitudes towards consent options for data sharing may not be a good proxy for the views of patients undergoing clinical testing was a motivation for understanding the level of control our patients wanted for the use of their clinical genomic data for research. The hypothetical levels of control we asked patients to choose between in our study are based on qualitative focus group discussion (Gleeson, unpublished). Direct comparators for these are lacking in the literature. A Swedish study recontacting biobanking participants about the use of their stored blood for genetic research offered similar levels of control. In comparison to the Swedish biobanking participants, twice as many of our respondents desired a choice each time [29]. A systematic review of biobank-based genomic research also noted differences between patient and public views on consent [5]. This lends support to our concern about the attitudes of the general public and biobank participants being used as a proxy for those of patients having clinical testing. The governance of clinical data systems and secondary use of patient data needs to consider work such as ours which reflects the attitudes of the patients whose data is being generated through clinical testing.

Australian national and state governments envisage a national approach to genomic information management, encompassing clinical and research-generated data [11]. This will need to consider the complex legislative environment in Australia [discussed in [30]] and potential differences in systems governing international data sharing [discussed in [31]]. Although some convergence between research and clinical care has been noted [discussed [32]], in most countries these activities continue to operate within distinct regulatory environments. As such, a clear delineation between the re-use of data for clinical and research activities is critical. In Australia, laboratories conducting genetic tests that have the potential to lead to complex clinical issues must collect evidence of consent, and this clinical test consent cannot be changed once a test is reported (although the patient may choose not to learn the results). In order for genomic data derived from clinical testing to be re-analysed in a research setting, a distinct research consent process is required [33]. The use of electronic ‘dynamic consent’ research platforms for this specific research consent process, as an *adjunct* to the clinical consent process, might meet differing patient needs with respect to the use of their clinical genomic data for research. They may also assist patients with recall of what they agreed which is desirable, as our results suggest recall diminishes over time.

Our study was designed to inform the sharing and re-use of genomic data files by clinical genetics laboratories. To our knowledge, it is the first to investigate patients who have decided whether to share their genomic data at the time of clinical testing. Survey response rates were high and our survey cohort largely reflected the diversity of our clinical cohort and included perspectives often missing in research studies, i.e., decliners and those with accessibility, literacy or language barriers. We do note, however, some limitations. There was variation in the number of respondents completing each question and survey respondents were more likely to be from higher income quartiles. In addition, we lack detailed qualitative comments from decliners that would illuminate the reasons for their choice.

We are uniquely positioned to highlight some specific considerations for those establishing clinical genomic data systems who wish to enable data sharing for secondary uses:

- information material should clarify who will *not* be able to access data. It was evident that some respondents held misconceptions about access to data by insurers and employers. Concerns about genetic discrimination are not unusual for people with genetic conditions [34]. Noting that legal requirements may require release in some circumstances, reassurance should be provided where possible.
- information about general security and privacy measures should be available. This might be most useful to those who fit with the ‘information seeking’ profile but further research is needed to determine what information would be useful and how it can best be conveyed.
- data sharing policies, procedures and information resources need to reflect diverse and specific attitudes regarding data sharing internationally. For instance, the USA was identified variously as both a desirable and an undesirable destination for data. Adherence to guidelines for researchers to conduct due diligence and risk assessments regarding international collaborations [35], where they exist, could address some - but not all - concerns. A standard approach that is both feasible and acceptable to patients needs to be developed through participatory research. Additional qualitative work would also be useful to further understand perceptions of international data sharing.
- decliners may benefit from further information and having their concerns addressed, particularly those of parents. Further understanding of mistrust, due to historical actions within the research community or experiences of persecution, may also assist engagement with patients from marginalised groups. However, we do not have sufficient open-text responses to elaborate on their needs. It remains to be determined to what extent this might change their decision.

The need for inclusive and equitable approaches to genomics is now widely recognised. Participants with English as an additional language were more likely to feel insufficiently informed, despite the use of interpreters in clinical consultations, as noted in other areas of healthcare [36]. Data-sharing decision-making may be impacted by the translation of information (e.g., the lack of appropriate translations for genomic vocabulary) and cultural differences [37]. Care must be taken that digital tools and other support for decision-making do not exacerbate inequalities or barriers to access. Investment of resources into appropriate support is needed and may be considerable in multicultural countries where there are many language groups in the community.

Our findings are highly relevant for the inclusion of clinical genomic data in international efforts to share data for research. Despite the high level of agreement to share their clinical genomic data, patients were not willing to give researchers free reign. The diversity of views on when patients considered data sharing to be risky and when it would be personally unacceptable suggests that data governance needs to both incorporate a level of patient control and be transparent about the safeguards, policies and processes in place. Without these, it is possible that a patient’s acceptance of clinical genomic testing could be undermined by their data-sharing concerns. That said, the willingness of patients to share their genomic data files - and the value of the resulting research - should encourage clinical services and laboratories to consider data sharing systematically for secondary uses. Further research – particularly at a national level – is required to move beyond understanding patient data-sharing attitudes to identification of good data governance (including policies, procedures and safeguards) which address use of clinically generated data for research and is informed by community and patient views. Rather than being seen as a risk to avoid, good data governance should be a challenge to rise to and should include engaging with patients to ensure data-sharing processes account for their values and preferences.

### Supplementary information

Supplementary Materials

## Data Availability

Additional data are available from the corresponding author on reasonable request.
