# Supplementary material for: Secondary use of genomic data: patients’ decisions at point of testing and perspectives to inform international data sharing
Source: Eur J Hum Genet. 2024 Mar 25;32(6):717–24. doi: 10.1038/s41431-023-01531-5 (PMC11153578; doi:10.1038/s41431-023-01531-5)
Supplement: Supplementary file 1 — Supplementary Materials [file 41431_2023_1531_MOESM1_ESM.pdf]

SUPPLEMENTARY MATERIAL

SUPPLEMENTARY FILE 1

Guide for those conducting consent conversations. Points to cover in discussion about sharing of genomic data for secondary use

**Point from consent form**	**Explanatory notes**
Anonymised data sharing	Patient should be aware this permits their data to be used in the ways described in section 6.1 of information sheet 1.<br>Patient anonymised data (identifiers removed when shared) can be used for the purpose of research and to understand natural variation in human genes, in Australia and internationally.<br>Encompasses permission for data being stored in online, restricted access, secure databases that meet international security and safety standards, in Australia and internationally.<br>Patient cannot expect to receive any personal results from such sharing, but their data may contribute to greater understanding generally, and could improve our ability to interpret variants.
Re-contact to obtain consent for use for other purposes	This permits the patient to be contacted for activities, including research, that are not related to the condition for which the test was originally ordered. Some examples include:<br>Clinical example 1: the patient may present with a second condition and may provide consent for their original genomic data to be re-analysed for the second condition (a new analysis for a different indication - a new consent form would need to be completed).<br>Clinical example 2: The patient may require a pharmacogenomic analysis to guide choice of medication. Even if this is a medication is for the condition for which the exome test was ordered, this would be a new request for reanalysis for a different purpose and may require a separate consent discussion.<br>Research example: Data in re-identifiable format for use in population research for an unrelated condition - the patient would need to be contacted before their data and healthcare information could be used for this purpose.
Consent to share DNA/data/information in a way that enables re-identification for return of results (if applicable)	This permits patients to opt for their data and health information to be used for activities, including research, related to their condition.

	If the patient agrees, the data can be shared in a manner that is re-identifiable so results can be returned.<br>Incidental findings should be minimal as the sharing of data is for the same purpose that the data was originally generated – to find the cause of the patient's condition.<br>Examples are specified in section 6.2 of Information Sheet 1 and at the end of the consent form (**Supplementary file 2**). A separate consent form may need to be signed for these activities. Examples include:<br>The ability to share genomic and phenotypic information with local and international platforms in order to find other patients with the same or similar phenotypes who also have variants in common.<br>Gene discovery research aimed at identifying the cause of their condition.<br>Functional studies (in a research or clinical setting) that may help provide data for or against pathogenicity of a variant identified in the patient.

SUPPLEMENTRAY FILE 2

Examples of data sharing provided at consent to patients, to support informed decision making about opting in or out of sharing their potentially identifiable information

Examples of how information that identifies you may be used

Some examples of how your or your relative's information could be used in a way that may identify you are listed below. These uses may reveal information that may be important to your health, or the health of your blood relatives for all the examples below, your information can be linked to you so any information with important health implications can be returned to you.

**Example of use:**
**Activities to clarify the cause or management of your condition.***<br>For example, your doctor may share your information with international groups trying to identify variants common to people who all have the same condition. The greater the number of patients with the same condition and the same variants, the more likely the variants are to contribute to the condition.

**Research to identify new genes associated with your condition**.*<br>Future, ethically approved research opportunities aimed at finding the cause of your condition may be offered. This may involve a re-analysis of your genomic data. With your permission, your doctor and the laboratory could share your information, meaning researchers may not need to re-sequence your genome and you would not need to provide another sample.
**Trying to clarify what a specific variant does in the cells of the body.***<br>To help clarify whether the variant affects cell functioning, the laboratory may ask for your information so they can do further work that may provide clues to the role of the variant.

*You may be asked to sign a separate consent form for this use.

SUPPLEMENTARY FILE 3

Survey respondent demographics and comparison of survey respondents to sample frame

Supplementary File 3, Table 1. Demographics of survey respondents (N = 1030)

Characteristics		N (%)
Gender	Female<br>Male	519 (50.4)<br>511 (49.6)
Respondent type	Adult patient<br>Parent of child patient	749 (73)<br>281 (27)
Assistance required to complete survey?	Yes<br>No	62 (6)<br>968 (94)
Condition type	Hereditary<br>Hematological/Malignant (Hem/Malignant)	620 (60)<br>410 (40)
Test type	Whole exome sequencing<br>Panel test	758 (74)<br>272 (26)
Country of birth (of patient)	Australia<br>Overseas<br>Unknown/missing	842 (82)<br>181 (17.5)<br>7 (0.5)
English as a first language	Yes<br>No<br>Unknown	883 (86)<br>109 (11)<br>38 (4)
Income[a]	Lowest quintile<br>Second quintile<br>Third quintile<br>Fourth quintile<br>Fifth quintile	206 (24)<br>120 (14)<br>171 (20)<br>201 (24)<br>148 (17)
State IRSAD classification[b]	Lowest quintile<br>Second quintile<br>Third quintile<br>Fourth quintile<br>Fifth quintile	166 (16)<br>139 (13.5)<br>168 (16)<br>233 (23)<br>324 (31.5)
Location[c]	Metropolitan Victoria<br>Regional Victoria<br>Interstate	749 (73)<br>226 (22)<br>55 (5)
Identify as Aboriginal or Torres Strait Islander	Yes<br>No<br>Unknown	6 (0.5)<br>1011 (98)<br>13 (1.5)

[a] Gross household income categories were re-categorized into quintiles in alignment with gross household income for Victoria for the 2015–16 financial year, as captured in the Australian Census, 2016 (Household Income and Income Distribution, Victoria. Australian Bureau of Statistics. Released 8 December 2017); 138 prefer not to say/missing responses are excluded.
[b]Index of Relative Socio-economic Advantage and Disadvantage (IRSAD), based on their postcode using data from Australian Bureau of Statistics Census 2016.
[c]Based on postcode and categorized into metropolitan Victoria, regional Victoria, or Interstate, as defined by Regional Development Victoria (www.rdv.gov.au).

After adjusting for gender, survey respondents are more likely to be adults who speak English as a first language from the two upper income quintiles.

Supplementary File 3, Table 2.

	**Odds Ratio**	**p**	**[95% Conf. Interval]**	
Gender - female	1.182	0.161	0.936	1.493
Respondent type - Adult	1.716	<0.0001	1.332	2.211
English as first language				
Yes	1.82	<0.0001	1.31	2.52
Income quintile				
21-40%	1.086	0.689	0.726	1.624
41-60%	1.082	0.685	0.739	1.583
61-80%	1.634	0.011	1.120	2.382
81-100%	1.573	0.013	1.098	2.252

SUPLLEMENTARY FILE 4

Open comments from those who declined to share their/their child's data for secondary use

Supplementary Table 4

**Respondent type**	**Condition type**	**Decision about data sharing**	**Sufficiency of Information**
Participant	Hereditary	maybe willing to depending on outcomes of results.	
Participant	Hereditary	If it is valuable and can help with finding a cause and a treatment, I would like to contribute what I can.	
Parent	Hereditary		Need more explanation
Parent	Hereditary		No matter how much information was given it's still never enough as no matter what it's still hard choice to make
Participant	Haematological		I hope there is a secure database to store all electronic records and that records are only accessible to appropriate personnel. I hope there is a well thought out records/info management system to avoid e.g. inappropriate distribution of personal information.<br>I hope all data/info collected is de-identified in final research report.
Parent	Hereditary	Sharing anonymous data only	
